# EXPERIENCES AND PERCEPTIONS OF FOREIGN NURSES WORKING IN A SUPER-AGED SOCIETY: A QUALITATIVE DESCRIPTIVE STUDY

**DOI:** 10.1093/geroni/igad104.3324

**Published:** 2023-12-21

**Authors:** Derong Zeng, Kyoko Asakura, Ayae Kinoshita, Bingbing Qi

**Affiliations:** Kyoto University, Kyoto, Kyoto, Japan; Tohoku University, Sendai, Miyagi, Japan; Kyoto University, Kyoto, Kyoto, Japan; Villanova University, Villanova, Pennsylvania, United States

## Abstract

This study delves into the experiences of foreign male nurses from China working within Japan’s super-aged society, a distinctive environment marked by unique healthcare and elderly care systems. We directed our focus on the Chinese perspective, aiming to gain a detailed understanding of their experiences, challenges, and perceptions within this context. This qualitative research explores experiences, challenges, and perceptions of 16 Chinese male nurses in Japan’s super-aged society. Focused on the Chinese perspective, it provides valuable insights via semi-structured interviews. The interviews were conducted in Chinese via Zoom, with each participant being interviewed once for an average duration of 90 minutes. Key findings include: 1) Job Responsibilities and Challenges: Participants reveal high work intensity and difficulties with disease management and scheduling in the Japanese healthcare system. 2) Aging Society Implications: While Japan’s eldercare and healthcare systems provide stability, they also result in increased demand and workloads. Some system aspects were criticized as unreasonable. 3) Cultural Adaptation: Challenges included language barriers, understanding Japanese etiquette, and lifestyle and work habits adjustment. 4) Interpersonal Relationships: Narratives about interactions within this new cultural environment highlighted communication issues and feelings of isolation. this study offers an in-depth understanding of the unique challenges faced by Chinese male nurses within Japan’s super-aged society. The findings serve to inform future healthcare policies and practices tailored towards foreign healthcare professionals in Japan. By focusing specifically on Chinese male nurses, this study contributes a fresh perspective to the ongoing discourse on cultural diversity within the international healthcare workforce.

